# Chemotherapy induces cell plasticity; controlling plasticity increases therapeutic response

**DOI:** 10.1038/s41392-023-01500-w

**Published:** 2023-07-03

**Authors:** Francisco J. Iborra, Cristina Martí, Virtu Calabuig-Navarro, Petros Papadopoulos, Salvador Meseguer, Pedro M. Iborra, Francisco García, Antonio Martínez-Lorente, Fernando Almazán, Juana Calabuig

**Affiliations:** 1 Centro de Investigación Príncipe Felípe (Associated Unit to Instituto de Biomedicína de Valencia), Valencia, Spain; 2 Pathology and Molecular Therapy Department, Instituto de Biomedicína de Valencia, (IBV), CSIC, Valencia, Spain; 3 Molecular and Cellular Biology Department, Centro Nacional de Biotecnología (CNB), CSIC, Madrid, Spain; 4 Hematology Department, Hospital Clínico San Carlos, Madrid, Spain; 5 Medical Systems Biology, Faculty of Medicine, University of Helsinki, Helsinki, Finland; 6 IC Biomed, Alicante, Spain; 7 Vinalopó Hospitals, Elche, and Biotechnology Department, Universidad de Alicante, Alicante, Spain

**Keywords:** Cancer therapy, Biophysics, Cancer therapy

**Dear Editor**,

One of the most serious issues in modern oncology is the ineffectiveness of treatments in destroying tumours, which leads to tumour recurrence and, ultimately, patient death. This phenomenon is caused by conventional therapies’ fractional killing of tumour cells, which can also cause resistant cells to spread.^1^

We analysed chemotherapy-resistant cells and discovered that they are smaller than untreated cells (Fig. 1a). The fact that this phenomenon occurs in all tested cell types (Hela, A549, Huh7, and MCF7) and regardless of the chemotherapy regimen (TRAIL, Camptothecin, Doxorubicin) suggests that it is a widespread phenomenon (Fig. 1a, Supplementary Fig. S1). Except for Doxorubicin, which causes cells to accumulate in G1 phase of the cell cycle, this reduction in the cell volume of resistant cells is not caused by selective cell death at a specific stage of the cell cycle (Fig. 1b). Nor is it due to the selection of small cells, as with all treatments we find a population of small cells that does not exist in untreated cells, gray area (Fig. 1a).

**Fig. 1:**
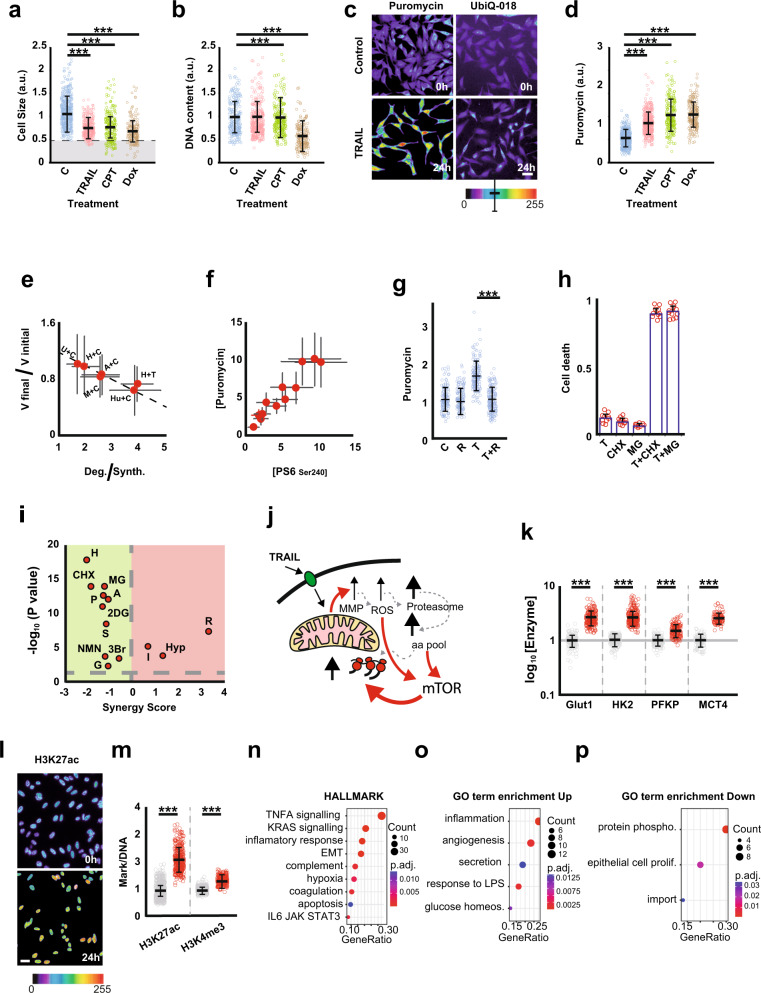
**a** Chemotherapy kills more big cells than small cells. The distribution of cell size after various treatments. Sizes were measured using the materials and methods section, and each point represents a single cell measurement. The distribution’s mean and standard deviation are shown in black. CPT stands for camptothecin, and Dox stands for Doxorubicin. Measurements were taken after 24 h of drug exposure, and values were normalised relative to control cells. The concentration of CPT is 10 μM, the concentration of TRAIL is 30 ng/ml, and the concentration of Dox is 2 μM. ***P < 0.001. **b** Only Doxorubici -induced size reduction can be attributed to G1 arrest. The amount of DNA was measured as the integrated intensity of the DAPI signal. Only Dox induced accumulation of cells in the G1 phase. ***P < 0.001. **c** Treatment-resistant cells possess higher protein synthesis and degradation activity than untreated cells. In this panel, we show representative images of proteasome activity using UbiQ-018, or puromycin incorporation as a reporter for protein synthesis activity. To enhance visualisation we use a pseudocolour palette (at the bottom of the panel). Bar 20 μm. **d** All treatments induce protein synthesis at short times. Analysis of puromycin incorporation in Hela cells 4h after the start of treatment, when no cell death events have yet been detected. measurements are standardised per individual cell. **e** The reduction in cell volume is a consequence of an imbalance in the protein synthesis/degradation balance in favour of the degradation processes. To generate this graph, we measured the protein degradation/synthesis balance by measuring protein degradation and protein synthesis at time 0 and 4h after starting treatment (Values obtained from Supplementary Fig. S2, see material and methods for details). In turn, for each treatment we measured the average volume at the beginning and at the end of the treatment. Legends: (A) A549, (H) Hela, (Hu) Huh7, (M) MCF7, (U) U2OS, (C) Camptothecin, (T) TRAIL. Thus, the higher the protein degradation/synthesis ratio, the greater the size reduction. The error bar corresponds to the calculated errors. **f** Treatments induce increased protein synthesis through mTOR activation. This panel shows the relationship between the concentration of PS6 Ser240, a reporter of mTOR activity, and puromycin incorporation. The concentration of these probes was calculated by dividing the intensity of the probes by the intensity of succinimidyl ester coupled with Alexa 647 (a reporter for measuring cell volume). To generate this graph we measured both parameters in single cells from different cell lines (Hela, A549, Huh7, MCF7), treated with TRAIL (30ng/ml), Camptothecin (10 μM) or Doxorubicin (2 μM). **g** The inhibition of mTOR activity abolishes the chemotherapy-induced increase in protein synthesis. Incubation with the mTOR activity inhibitor rapamycin inhibits the TRAIL-induced increase in protein synthesis. In this experiment, we used Hela cells that we exposed to TRAIL (30 ng/ml) (T), Rapamacyn (1 μM) (R), to the combination of both (T+R) or to nothing (C). **h** Protein synthesis and degradation activities are important in conferring resistance to chemotherapy. Cells were exposed for 6h to TRAIL 7.5 ng/ml (T), Cycloheximide 50 μg/ml (CHX), MG132 at 5 μM (MG), or combinations of TRAIL and CHX or TRAIL and MG132. **i** Synergy analysis of drug combinations for apoptosis induction. For this panel, the synergy score was obtained as described in material and methods. A negative synergy score means an increase in cell death when compared with TRAIL alone (green region). A positive Synergy score means inhibition of apoptosis when compared with TRAIL alone (pink area). Following the analogy with volcano plots, we display synergy score versus the probability associated. This plot shows that drugs which inhibit translation or proteasome are increasing cell death. HomoHarringtonin (H) (50 nM, 10h), cycloheximide (CHX) (50 μg, 10h), puromycin (P) (1 μg, 10h), anisomycin (A) (100 nM 6h), MG132 (MG) (5 μM 12h). While, activation of the proteasome, Rapamycin (R) (1 μM, 12h), inhibits apoptosis when incubated with TRAIL. Glycolysis is needed for cell survival after TRAIL treatment. Inhibition of glycolysis activates synergistically apoptosis by TRAIL. 3-Bromopyruvate (3Br) (30 μM, 10h); 2-Deoxyglucose (2DG) (25 μM, 6h); Shikonin (S) (4 μM, 10h). Idebenone (I) (2 μM, 12h), which bypass complex I inhibition, increases cell survival. In addition, the induction of stress such as hypoosmotic (Hyp) stress (0.75XDMEM) that produces increased protein synthesis and degradation makes cells more resistant to TRAIL. **j** Diagram summarizing the mechanism of proteostasis induction by TRAIL at short times. The apoptotic cascade is started when TRAIL binds with its receptor on the plasma membrane. After becoming active, Bax attaches to the mitochondria and inhibits complex I of the electron transport chain. This process causes the mitochondria to become hyperpolarized, which leads to the production of free radicals that activate the proteasome. This final step causes a rise in the intracellular amino acid concentration, which stimulates protein synthesis. Both free radicals and the increase in free amino acids could be responsible for the activation of mTOR. **k** TRAIL increases the glycolytic flux of cells after 24h exposure. Analysis of the changes in the concentration of the enzymes controlling the glycolytic flux. Gray circles values of concentration in control cells, in red, TRAIL (30 ng) treated cells. **l** TRAIL induces epigenetic modifications. Micrograph showing the staining of histone H3K27 acetylation, a chromatin modification associated to RNA pol II elongation. After 24h pf TRAIL treatment, surviving cells show an increase in H3K27acc. Bar 20 μm. **m** Quantification of images like the displayed in panel m normalized by the DNA content of each individual cell. We measured H3K27ac and H3K4m3, both modifications associated with cellular plasticity. Then, this analysis shows that TRAIL treatment induces epigenetic reprogramming of surviving cells. Gray circles values of concentration in control cells, in red, TRAIL (30 ng) treated cells. **n** Transcriptomic analysis of the surviving cells to the different treatments. Functional analysis of the genes induced by the three treatments. Hela cells treated for 24 h with TRAIL (30 ng/ml), Camptothecin (10 μM), or Doxorubicin (2 μM), all of which killed around 50% The Hallmarks functional analysis show enrichment in diverse functions such as inflammation and stress responses. **o** Functional analysis of the 46 upregulated genes (Supplementary table S2) in the small cells that have not been exposed to the drugs. **p** Functional analysis of the 31 downregulated genes (Supplementary table S3) in the small cells prior to treatment

The alteration in the biosynthesis and/or degradation of proteins, which are the macromolecules that contribute most to cell size, may therefore be the cause of the shrinkage. We then analyzed protein synthesis using puromycin, which is incorporated into nascent proteins, and UbiQ-018, which is a fluorescent reagent used to measure proteasomal activity. Hela cells resistant to TRAIL treatment (24 h, 30 ng/ml) have a higher concentration of protein synthesis activity, as well as higher proteasomal activity than untreated cells (Fig. 1c). We measured proteostatic activity (protein synthesis and degradation) 4 h after starting the treatments, when cell death had not yet occurred. The treatments used in this study increased protein synthesis (Fig. 1d). For degradation studies we focused on the effect of camptothecin which, like translation, increases activity (Supplementary Fig. S2). Changes in the protein synthesis/degradation balance caused by camptothecin correlate with the observed cell volume variation (Fig. 1e), supporting the hypothesis that cell size changes are caused by a change in the proteostatic balance.

Protein synthesis may be regulated at multiple levels. The mTOR pathway impacts translation. Phosphorylation of ribosomal protein S6 at Ser240 (PS6) is a target of mTOR and modulates protein synthesis. Treatments increased PS6 levels that correlate with changes in puromycin incorporation (Fig. 1f). Furthermore, when we inhibited mTOR with rapamycin (1 μM) exposure to TRAIL 30 ng did not induce changes in translational activity. These results altogether suggest that the increase in protein synthesis after treatments is a consequence of mTOR activation (Fig. 1g).

Next, we investigate whether high proteostatic activity (protein synthesis and degradation) is required for surviving apoptotic signals. When cells were treated with TRAIL or camptothecin as well as translation inhibitors, a strong synergistic effect was observed (Fig. 1h, Supplementary Fig. S3). Similarly, proteolysis appears to be important for chemotherapy resistance, as when cells were incubated with TRAIL or Camptothecin in the presence of MG132, a synergistic effect was observed (Supplementary Fig. S3). Similar behavior was observed in A549 or Huh7 cell lines, suggesting that it is a widespread phenomenon (Supplementary Fig. S4).

On the other hand, cells became TRAIL-insensitive after we induced proteolysis by incubating them with low micromolar concentrations of Rapamycin (Fig. 1i).

We also investigated the role of protein turnover modulation in apoptosis.

Allowing Hela cells to grow in a glucose-free medium (using glutamine as an energy source) or stressing cells (hypotonic stress 0.75% PBS for 4 h) increased protein translation and protein degradation activity concentrations. When Hela cells were starved of serum for three days, the same effect was observed. In every case, the cells developed resistance to TRAIL (Supplementary Fig. S3). Multinucleated cells, on the other hand, were extremely sensitive to TRAIL and showed reduced translational and proteolytic activities that did not exceed control levels (Supplementary Fig. S3). These findings support the existence of a critical protein turnover threshold for apoptosis induction.

So, the increase in protein turnover would be a cellular plasticity mechanism that allows for treatment adaptation.

To gain insight into the dynamics of the plastic response to chemotherapeutic treatment, we studied the physiological changes that occurred in the cells after exposure to TRAIL for different periods of time.

After TRAIL binds to its receptor on the plasma membrane, the apoptotic cascade begins. After that, Bax becomes activated, binds to the mitochondria, and inhibits complex I of the electron transport chain^2^ (Supplementary Fig. S5). This process causes the mitochondria to hyperpolarize (Supplementary Fig. S5), releasing free radicals that activate the proteasome (Supplementary Fig. S5). This final step raises the intracellular amino acid concentration, which stimulates protein synthesis. This process seems to be mediated by mTOR signaling (Fig. 1j). This mechanism appears to be common; it has already been observed in oculopharyngeal muscular dystrophy, where proteasome hyperactivation occurs as a result of increased ROS production caused by mitochondrial respiratory dysfunction, which results in cell contraction.^3^

Complex I inhibition causes an increase in glycolytic flux to compensate for the ATP deficit caused by electron transport chain inhibition.^4^ The increased expression of Glut1, HK2, PFKP, and MCT4, the four key steps controlling glycolytic flux,^5^ demonstrates this (Fig. 1k). Glycolytic transformation is important for chemotherapy resistance because inhibiting this process with 2-DeoxyGlucose, 3-Brpyruvate, or Shakonin has a synergistic effect with TRAIL, Idebenone which bypasses Complex I protects cells (Fig. 1k).

Therapy, also causes epigenetic changes in chromatin. After 24 h of TRAIL treatment, two chromatin activation epigenetic markers, H3K4me3 and H3K27ac, increased (Fig. 1l, m), supporting transcriptional reprogramming.

Following that, we examined the transcriptomes of Hela cells treated for 24 h with TRAIL (30 ng/ml), Camptothecin (10 μM), or Doxorubicin (2 μM), all of which killed ~50% of the cells. These treatments induced the expression of 208 genes that they all had in common. Transcriptome functional analysis revealed an enrichment in TNFA, KRAS, inflammatory response, mesenchymal epithelial transition, complement, hypoxia, coagulation, apoptosis, and IL6, JAK, and STAT3 signalling, which is consistent with the stress response (Fig. 1n). This viewpoint is supported by the induction of stress-related transcription factors (ETV1, RELB, FOSB, NFATC1, FOS, FOSL1, ETV4 or JUN). As a result, the cell’s transcriptome has changed in response to chemotherapy-induced stress, resulting in a more plastic phenotype, evidenced by the induction of Snail1 expression (Supplementary Fig. S7), this increases its ability to survive in harsh environments.

The two phases of phenotypic plasticity are detection and adaptation to environmental changes.

The treated cells show signs of both phases. First, resistant cells express more internal (RIGI) and external signal transduction-related genes (TNFRSF1B, TNFRSF12A, IL4R, TNFRSF10A, TLR4, IL17RD, TNFRSF21, FCMR, TGFBR2, IL7R, IRS1, PTGER4, FZD8, TNFSF15, GPR3, ITGB3, FGFR1, PLEK2, HBEGF, RGS2). In addition, we discovered genes involved in evasion and transformation throughout the response phase (Table S1, Supplementary Fig. S7).

Since chemotherapy causes a reduction in cell size, and also induces cell plasticity, we investigated whether plasticity and cell size are related. When we compared the transcriptomes of small and big cells, we observed that small cells expressed more EMT genes (Supplementary Fig. S7), but they also had a higher capacity to repair damaged DNA, more signalling noise, and transcription noise, all of which are associated with cellular plasticity (Supplementary Fig. S6). Suggesting that small cells are more plastic than big cells.

To further understand how cells, alter the plastic response to chemotherapy, we analysed the expression of these 208 induced genes in small and big cells that had not received treatment. 46 genes were up-regulated and 30 were down-regulated in small cells (Fig. 1o, p; Table S2, S3). These gene sets’ GO term analyses showed that up-regulated small cell genes like IER3, RelB, JUN, FOS, or GATA6 were associated with the acute stress response. Genes that were down-regulated were enriched in cell signaling-related processes. Small cells are so primed to react to rapid stress, and later in the adaptation process, they will start expressing genes involved in cell signalling.

Chemotherapy, according to our findings, causes early oxidative stress (Supplementary Fig. S5c), triggering a sustained stress response. As a result, the cell is on high alert, with most signalling pathways active as an immediate line of defence. This gene expression reprogramming configuration would make these cells virtually invincible.

Targeting phenotypic plasticity would be an excellent strategy to curtail both metastasis and therapy escape.

Our findings indicate that targeting protein synthesis and degradation during the response phase would be a more effective approach, as this would prevent phenotypic reprogramming and, as a result, resistance development.

The findings of this study suggest that cell size influences cellular plasticity and, as a result, tumour aggressiveness. Our findings sustenance the long-held observation that tumour aggressiveness and the size of small tumour cells are related.

## Supplementary information

Supplenetary Material

## Data Availability

RNAseq data have been deposited in the NCBI database under accession code BioProject ID: PRJNA416451, and GSE22976.

## References

[1] Karagiannis GS, Condeelis JS, Oktay MH (2019). Chemotherapy-induced metastasis: Molecular mechanisms, clinical manifestations, therapeutic interventions. Cancer Res..

[2] Kim EM (2014). Nuclear and cytoplasmic p53 suppress cell invasion by inhibiting respiratory Complex-I activity via Bcl-2 family proteins. Oncotarget.

[3] Ribot C (2022). Activation of the ubiquitin-proteasome system contributes to oculopharyngeal muscular dystrophy through muscle atrophy. PLoS Genet..

[4] Yang M (2021). Inhibition of mitochondrial function by metformin increases glucose uptake, glycolysis and GDF-15 release from intestinal cells. Sci. Rep..

[5] Tanner LB (2018). Four key steps control glycolytic flux in mammalian cells. Cell Syst..

