# Supplementary material for: Chemotherapy induces cell plasticity; controlling plasticity increases therapeutic response
Source: Signal Transduct Target Ther. 2023 Jul 3;8:256. doi: 10.1038/s41392-023-01500-w (PMC10315372; doi:10.1038/s41392-023-01500-w)
Supplement: Supplementary file 1 — Supplenetary Material [file 41392_2023_1500_MOESM1_ESM.docx]

Supplementary Materials for

Chemotherapy induces cell plasticity; controlling plasticity increases therapeutic response.

**Authors:** Francisco J Iborra^;^ Cristina Martí; Virtu Calabuig-Navarro; Petros Papadopoulos; Salvador Meseguer; Pedro M Iborra; Francisco García; Antonio Martínez-Lorente; Fernando Almazán and Juana Calabuig.

*Correspondence to: [fjiborra@ibv.csic.es](mailto:fjiborra@ibv.csic.es); [fjiborra@cipf.es](mailto:fjiborra@cipf.es)

**This PDF file includes:**

Materials and Methods

Supplementary Text

Figures. S1 to S7

Tables S1 to S3

**Material and methods:**

Cell lines and materials.

Huh7, A549, MCF7, U2OS and HeLa (ATCC CCL-2) cells were grown in Dulbecco’s Modified Eagle Medium (DMEM, Gibco)–GlutaMAX-I supplemented with 10% foetal bovine serum (FBS, Hyclone) and penicillin–streptomycin (Sigma) in a 37 °C humidified incubator with ~5% CO2. For particular experiments DMEM without glutamine or without FBS was used.

Mitochondrial mass for in vivo experiments was measured as the integrated signal of MitoTracker Green FM (MG, Molecular Probes) incorporated by individual cells. The apoptotic signal was triggered by human recombinant TRAIL (Millipore) at the indicated dosses.

TRAIL, Rapamycin, BCH, HomoHarringtonin, Idebenone, Cycloheximide, Puromycin, Anisomycin, MG132, CX5461, Leptomycin B, 2DeoxyGlucose, Shikonin, otinamide Mono Nucleotide (NMN) and 3BrPyruvate were purchased Sigma Aldrich. UbiQ018 from UbiQbio, the Nederland’s

Cell volume determination

To determine the cell volume, we use a method previously described by^1^. The total protein content is determined after total protein staining using succinimidyl ester coupled with Alexa 647 (Succ) (Invitrogen). This dye makes covalent bonds with proteins on primary amines. After immunolabelling the cells were incubated with Succ 0.2μg/mL in PBS for 5 minutes. Then cells were washed and mounted on coverslips. After ~~which~~ cell ~~were imaged~~imaging the integrated signal intensity was calculated as the product of net intensity times area of the cell. The amount of signal coming from individual cells is proportional to cell volume ^1^. At least 500 cells were measured for each case.

Cell Immunostaining (Wide-confocal Cytometry)

HeLa cells growing on coverslips were fixed and proteins indirectly immunolabelled using a 1:500 dilution of the corresponding primary antibodies. The fixation was performed for 10 min at RT with 4% PFA and the permeabilization, for 10 min with 1% Triton X-100 in PBS. Secondary antibodies were Alexa Fluor 488 or 546 donkey anti-mouse, goat, or rabbit IgG (H+L) (Invitrogen). Nuclei and cell volume were simultaneously labelled for 10 min with 6 nM of 4’,6-diamidino-2-phenylindole dihydrochloride (Dapi), and 10 nM of Succ-647, respectively. Coverslips were mounted in Vectashield (Vector Laboratories). Images of labelled cells were collected from a widefield Leica microscope DMi8 with an HCPL Fluotar L20X/0.40 PH1. The images were analyzed using the MetaMorph 7.8.0.0 software (Molecular Devices). At least 300 cells were analyzed for each antibody and condition assayed.

The protein antibodies were used at dilution 1:500 and were purchased from: EDM Millipore: Glut 1 (07-1401), Puromycin (MABE343); Cell Signaling Technology: Jun B (3753S), c-Jun (165T), FosB (2251), FRA1 (5281), PFKP (12746), Phospho-S6 (Ser240/244), HK2 (2867); Thermo Fisher: MCT4 (PA554413); Gene Tex: SNAIL1 (GTX125918). Caltag: IdU/BrdU (MD5010).

Puromycin incorporation

Hela cells grown in DMEM were pulsed for 5 minutes with 90 μM Puromycin (Sigma). Then, cells were fixed as described in the previous section and processed for immunostaining.

BrU incorporation

Hela cells grown in DMEM were pulsed for 30 minutes with 1mM BrU (Sigma Aldrich). Then, cells were fixed as described in the previous section and processed for immunostaining.

Quantification of the relative concentration of proteins and translation in individual cells.

We measured this parameter using the images of the fluorescence channel used to detect the antibody of interest and the image of the total protein content. Both images were obtained by widefield microscopy and quantified as described in Iborra and Buckle 2008^2^. The concentration of the protein of interest was calculated as the ratio of the integrated intensities of both channels.

Calculation of protein synthesis and degradation rate ratios.

For the calculation of the protein synthesis rate ratio in the treated cells, we proceeded as follows. At 0 and 4h after treatment the cells were pulsed with puromycin (90 μM) for 5 min, after which they were fixed and processed to detect puronylated proteins. Once the puromycin-incorporated proteins were immunodetected, the integrated intensity (net intensity times area) was quantified for both conditions (0 and 4h). The ratio of protein synthesis rates was calculated as the quotient of the integrated intensities at 4 and 0h. For the calculation of degradation rate ratios, cells treated at 0 and 4h were pulsed with UbiQ018 (2 μM) for 20 min. After incubation, cells were fixed with 4% paraformaldehyde, mounted on slides and visualised. We quantified the integrated intensity (net intensity times area) for both conditions (0 and 4h). The ratio of protein degradation rates was calculated as the quotient of the integrated intensities at 4 and 0h. The data generated with this approach are shown in supplementary Fig S2. Cell volumes were calculated after fixing cells at 0 and 4h treatment as described in the Cell volume determination section. Figure 1e shows the relationship between changes in the rate of protein degradation/synthesis and changes in cell volumes for the conditions described in the figure.

Cell sorting, RNA preparation, RNAseq, and data processing

HeLa cells were stained with Mitotraker Green (MG) for 40 min in DMEM. After the staining, the cells were washed twice with PBS, trypsinized, and resuspended in PBS with 5 mM EDTA. Then, the cells were sorted on a fluorescence-activated cell sorter MoFLo XDP (Beckman Coulter) into two populations of 10^6^ cells with high and low mitochondrial content with a difference in mitochondrial mass of around 5-fold. After a DNase treatment, total RNA from sorted cells was extracted using RNeasy Mini Kit (QIAGEN) according to the manufacturer's guidelines. The quality of the extracted RNA was measured by the RNA Integrity Number (RIN) value from Bioanalyzer (being in all cases higher than 8). Finally, 3 μg of purified RNA was sent to RNA-sequencing at the SNP&SEQ sequencing facility (Science for Life Laboratory (SciLifeLab), Uppsala sequencing node). Total RNA was depleted from rRNA before library construction. One lane per sample was used in a 60-bp paired-end run on an Illumina HiSeq 2500 sequencer. For each sample, over 50 million pair-end reads were sequenced. RNAseq data have been deposited in the NCBI database under accession code BioProject ID: PRJNA416451. The reads were aligned using STAR.2.6.1^3^.

For treated cells 24h after drug exposure RNA was extracted using RNeasy Mini Kit (QIAGEN) according to the manufacturer's guidelines and processed for sequencing. The total RNAs were sent to Center for Biomics Erasmus MC, Rotterdam, The Netherlands. The sequencing libraries were prepared using the Truseq stranded mRNA library preparation method from Illumina. These libraries were subsequently sequenced on an Illumina NextSeq2000 sequencer. Paired-end clusters were generated of 150 bases in length (BioProject ID: Pending of assignation). The reads were aligned using HISAT2 ^4^.

In both cases, the reference genome used was GRCh38. Annotation and quantification was performed using HT-seq count^5^.

After alignment and transcript quantification, all downstream analysis was performed with R software. For cells with high and low mitochondrial content, raw count normalization, filtering out low expressed genes and differential expression analysis was performed using the edgeR  package^6^ and the pipeline for paired samples. And for drug treated cells we use edgeR package to filter and normalise the data, followed by the **limma** package to assess gene differential expression^7^.

Genes with False Discovery Rate (FDR) ≤ 0.05 and at least a 1.4-fold change in expression (abs(log2FC) ≥ 0.5) were considered to be differentially expressed. Functional enrichment analysis (ORA or GSEA) was performed using clusterProfiler package^8^ with MSigDB ([https://davislaboratory.github.io/msigdb](https://davislaboratory.github.io/msigdb).)  for gene sets Molecular Signatures Database. The enrichplot R package ([https://yulab-smu.top/biomedical-knowledge-mining-book/](https://yulab-smu.top/biomedical-knowledge-mining-book/).) was also used to visualize the results.

RNAseq data have been deposited in the NCBI database under accession code: https://www.ncbi.nlm.nih.gov/geo/query/acc.cgi?acc=GSE229761

Synergy analysis.

The drug-drug synergy analysis was carried out using the Bliss independence test^9^. For this purpose, we analyzed the survival of cells at specific times used individually and in combination. We compared the survival values of the combined treatments with the theoretical ones if both drugs were independent. We defined the synergy score as the Log2 of the survival ratio between the experimental and theoretical values. Negative values of the synergy score mean a positive synergistic effect. While a positive value means that both drugs are antagonistic.

Mitochondria function, respiration and H_2_O_2_ production.

Oxygen consumption rate (OCR) and the rate of mitochondrial hydrogen peroxide production in mock- or TRAIL-treated HeLa cells were simultaneously measured using a high-resolution respirometer (Oxygraph-2 k, Oroboros Instruments, Innsbruck, Austria) together with the O2k-Fluo LED2-Module. In brief, 80%-confluent cells were detached at 37ºC with trypsin-EDTA, resuspended in fresh growth media and counted. 1.5x106 cells of mock or TRAIL-treated was simultaneously analyzed in two 2 mL-Oxygraph chambers. The rate of mitochondrial H2O2 production was evaluated using Amplex Red (10 µM, Thermo Fisher Scientific), horseradish peroxidase (HRP, 1 U/ml) and superoxide dismutase (SOD 5 U/ml) based on a previously described method ^10^. The reduction of hydrogen peroxide is coupled with the oxidation of Amplex UltraRed to resorufin. Resorufin fluorescence (excitation wavelength 563 nm and emission 587 nm) is converted to hydrogen peroxide concentration by using a hydrogen peroxide calibration curve obtained from freshly prepared hydrogen peroxide standards. The change of the emitted fluorescence intensity during addition of the cells, the mitochondrial substrates or inhibitors for the different mitochondrial respiratory complexes is directly proportional to the production of hydrogen peroxide. Substrates and inhibitors were sequentially added as follows: 5 mM pyruvate, 5 nM oligomycin (to inhibit complex V to assess non-mitochondrial respiratory capacity or leak rate), carbonyl cyanide-p-trifluoromethoxyphenylhydrazone (CCCP) uncoupler with stepwise titration in 0.5 mM increments (to assess maximal electron transport system respiratory capacity rate), 0.5 mM rotenone (to inhibit complex I), 10mM succinate (to evaluate whether there are cells with damaged cytoplasmic membranes) and 2.5 mM antimycin A (to inhibit complex III). Data were analyzed using DatLab7 (Oroboros, Austria) software.

**Figure S1**.

Analysis of cell size distribution at 24h of treatment. In all tested lines (A549, MCF7, Huh7) a similar size reduction to that observed in Hela cells was observed (Fig 1a) TRAIL 30 ng/ml; CPT 10 μM; Dox 2 μM. *** P <0.001.

**Figure S2**.

Analysis of protein synthesis and protein degradation in different cell lines at 4h of exposure to chemotherapy. In all the cases studied, we observed an increase in the incorporation of puromycin 4h after starting treatment. However, the increase in proteasomal activity (UbiQ-018) in all cases was greater than the increase in protein synthesis. This unbalanced the balance of protein synthesis and degradation towards the degradative processes. * P <0.05; ** P <0.005; *** P <0.001.

**Figure S3**. Protein synthesis and degradation activities are important to confer resistance. **(a)** Cells were exposed up to 6h to Camptothecin 5 μM (CPT), Cycloheximide 50 μg/ml (CHX), MG132 at 5 μM (MG), or combinations of CPT and CHX or CPT and MG132. **(b)** Kinetic analysis of the Translation after exposure to 5 μM CPT. **(c)** Kinetic analysis of the protein degradation (UbiQ-018). **(d)** Perturbations of proteostatic activity have an impact on the anti-apoptotic response. In this experiment, we adapted Hela cells to grow in different conditions that impact protein synthesis and proteolytic activity. In blue incorporation of puromycin and in red proteolytic activity. Multinucleation, both natural and induced by cell fusion (Mn), generates cells with a low concentration of proteostatic activity. Whereas, adaptation to living in a glucose-free environment, using glutamine as the main energy source (Gln), activates both translation and protein degradation. Exposure to 1 μM Rapamycin (R), increases protein turnover. The highest induction of these activities was found in cells adapted to live in a complete medium but lacking serum (SS). These cells were cultured for 72h.

**(e)** Cells with low proteostatic activity are the most sensitive and those with high activity are the most resistant. This experiment allows us to propose the existence of a proteostatic threshold that determines cell behavior towards apoptotic signals.


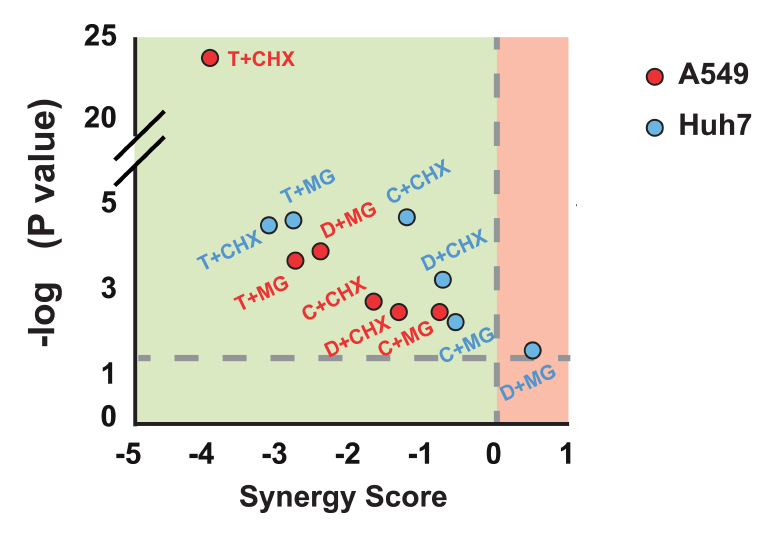


**Figure S4**. Synergy analysis of drug combinations for apoptosis induction in different cell lines. For this panel the synergy score was obtained as described in material and methods. A negative synergy score means an increase in cell death when compared with TRAIL alone (green region). A positive Synergy score means inhibition of apoptosis. This analysis demonstrates that inhibition of both protein synthesis and degradation exerts a synergistic effect on cell death of the drugs (T) TRAIL, (C) Camptothecin and (D), Doxorubicin in Huh7 and A549 cell lines. (CHX) Cycloheximide 50 μg/ml, (MG) MG132 5 μM.

**Figure S5**.

Analysis of the kinetics of stress response activation by TRAIL. **(a)** TRAIL induces mitochondria membrane hyperpolarization, measured as TMRM incorporation. **(b)** Oxygen consumption rate (OCR). TRAIL affects respiration at short incubation times. B stands for basal respiration; ATP stands for ATP linked respiration; PL stands for proton leak; RC stands for reverse capacity; M stands for Maximal respiration; nM stands for non-mitochondrial oxygen consumption. Notice that after 4h of TRAIL incubation ATP production is below 50%. **(c)** TRAIL induces free radical production. Quantification of reactive oxygen species using the reporter CellROX. TRAIL induces increase in CellROX signal per cell. **(d)** Rate of mitochondrial hydrogen peroxide. Oxidative Phosphorylation Complex I inhibition by TRAIL is responsible of ROS. As evidenced by the sensitivity to Rotenone (R). For this experiments TRAIL was used at 30 ng/ml. (B) stands for basal; (P) stands for pyruvate, (O) stands for oligomycin, (U) stands for CCCP uncoupler. (S) stands for succinate. **(e)** TRAIL treatment increases the proteasomal activity. Kinetic analysis of protein degradation activities. TRAIL induces an increase in the signal of UbiQ018, a reporter for proteasomal activity. Cells were incubated for 30 min with the compound after TRAIL incubation. Bar 20 μm. **(f)** TRAIL treatment increases the proteasomal activity. Quantification of images like the displayed in panel e, each point is a measure of one individual cell.

In summary. The apoptotic cascade is started when TRAIL binds with its receptor on the plasma membrane. After becoming active, Bax attaches to the mitochondria and inhibits complex I of the electron transport chain. This process causes the mitochondria to become hyperpolarized, which leads to the production of free radicals that activate the proteasome. This final step causes a rise in the intracellular amino acid concentration, which stimulates protein synthesis. Color code: gray is control; blue is 2h of TRAIL; pink is 4h of TRAIL, and red 24h of TRAIL. * P <0.05; ** P <0.005; *** P <0.001.


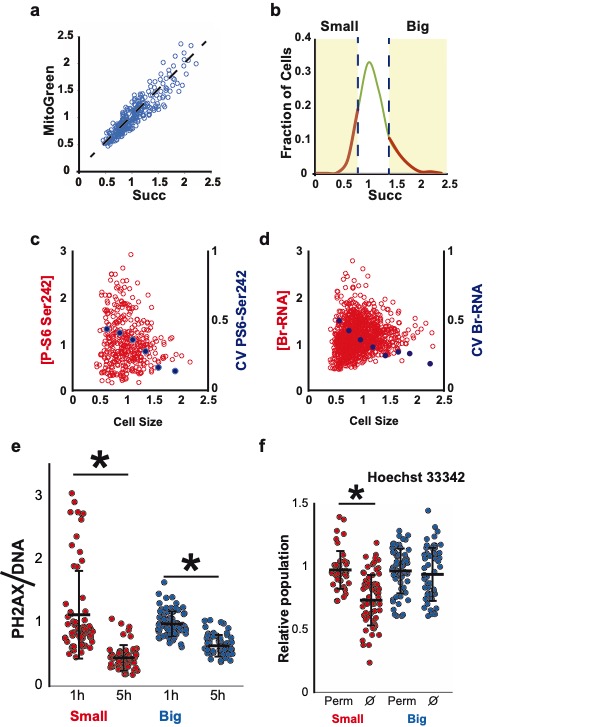


**Figure S6**. Small cells are more plastic than big cells. In this figure, we show several markers associated with cell plasticity, which are higher in small cells than in large cells. **(a)** As the marker used to measure cell volume (succ) can interfere with transcriptomic analysis, we explored the use of a surrogate size marker. In this panel, we show the correspondence between cell size and mitochondrial content (which is compatible with transcriptomic analysis). This analysis validates the use of MitotrackerGreen as a size marker. **(b)** Plot showing the size distribution of the Hela cell population used in the study. In this population, we selected small and large cells (marked in yellow), these cells represent 20% of the smallest cells and 20% of the largest cells. Therefore, we sort cells with low mitochondria (20% lower) and cells with high mitochondrial content (20% upper) for transcriptomic analysis. **(c)** Distribution of the P-S6 marker Ser242 in individual cells versus cell size (in red). This modification is associated with mTOR signalling. Small cells show a larger range of intensities than large cells. In the blue, right axis, we show the coefficient of variability (CV), which is the standard deviation divided by the mean in stratified samples (every 0.2 units of cell size). As can be seen (blue circles) the variability decreases as the cells get bigger. **(d)** Analysis of the variability of transcriptional activity versus size. Cells were exposed to BrU, which is incorporated into nascent RNA, for 30 minutes. As in the case of P-S6 Ser242, small cells show greater variability than large cells. In the blue, right axis, we show the coefficient of variability (CV). **(e)** Small cells have a higher DNA repair capacity than large cells. Hela cells were exposed to 6Gy of gamma radiation and fixed after 1 h or 5 h of treatment. In both samples (1 h and 5 h) we stained the cells with PH2AX, a DNA double-strand break marker, and quantified the signal, normalized to each cell’s DNA content. Small cells reduced the damage more than big cells, which is evidence of the existence of a more efficient repair system in these cells than in big cells. **(f)** In this assay (side population) we measured the exclusion capacity of the Hoechst 33342 dye. We measured the amount of dye incorporated in live cells (O) versus the amount incorporated in fixed and permeabilized cells (Perm). This plot shows that small cells have higher multidrug transporter activity than big cells. * P <0.001.

**Figure S7**.

Validation of gene expression induction by chemotherapy.

We selected several genes that were commonly overexpressed for the treatments used in our transcriptomics results in Hela cells. To see if the data obtained in our transcriptomic analysis had a broad spectrum, which did not only occur in Hela, we analysed the same genes in the Huh7 and A549 cell lines. As can be seen, the qualitative behaviour was identical for all genes and cell lines with the various treatments. It is important to note that genes involved in Mesenchymal Epithelial Transition (EMT) are strongly induced. This last result supports the main conclusion of this manuscript: chemotherapy induces cellular plasticity. * P <0.001 with their respective control.

**Table S1**.

List of genes upregulated by the three treatments.

| **Row.**  **names** | **ensembl_ID** | **logFC.**  **T vs C** | adj.P.Val.  T vsC | **logFC.**  **CPT vs C** | adj.P.Val.  CPT vs C | **logFC.**  **Dox vs C** | adj.P.Val.  Dox vs C |
| --- | --- | --- | --- | --- | --- | --- | --- |
| **CXCL8** | **ENSG00000169429** | **3,854** | 3,227E-08 | **5,564** | 2,432E-12 | **4,743** | 5,56E-11 |
| **ETV5** | **ENSG00000244405** | **3,522** | 1,989E-10 | **3,177** | 2,485E-11 | **2,942** | 1,54E-10 |
| **SPRY4** | **ENSG00000187678** | **3,389** | 7,056E-06 | **5,15** | 4,21E-10 | **4,228** | 1,871E-08 |
| **CXCL1** | **ENSG00000163739** | **3,137** | 9,605E-09 | **3,013** | 7,031E-10 | **2,488** | 2,912E-08 |
| **DUSP6** | **ENSG00000139318** | **3,087** | 1,008E-08 | **5,108** | 5,547E-14 | **4,943** | 1,897E-13 |
| **DUSP4** | **ENSG00000120875** | **2,914** | 7,275E-11 | **3,95** | 7,055E-15 | **4,309** | 5,699E-15 |
| **GPR3** | **ENSG00000181773** | **2,874** | 1,528E-06 | **4,866** | 1,112E-11 | **2,419** | 2,307E-06 |
| **IL6** | **ENSG00000136244** | **2,769** | 1,264E-08 | **2,908** | 1,907E-10 | **4,48** | 1,891E-13 |
| **ETV4** | **ENSG00000175832** | **2,672** | 1,866E-08 | **2,52** | 1,941E-09 | **1,942** | 1,767E-07 |
| **PHLDA1** | **ENSG00000139289** | **2,553** | 4,206E-12 | **2,593** | 4,225E-14 | **1,525** | 5,868E-10 |
| **TNFSF15** | **ENSG00000181634** | **2,539** | 7,854E-10 | **4,944** | 2,724E-16 | **4,544** | 3,126E-15 |
| **ITGB3** | **ENSG00000259207** | **2,438** | 1,389E-07 | **0,852** | 0,0061497 | **2,178** | 6,571E-08 |
| **NGF** | **ENSG00000134259** | **2,422** | 0,0011145 | **4,359** | 2,918E-08 | **5,39** | 9,303E-10 |
| **SDR16C5** | **ENSG00000170786** | **2,398** | 0,0054864 | **5,732** | 4,704E-09 | **5,542** | 1,079E-08 |
| **SPRY2** | **ENSG00000136158** | **2,344** | 2,523E-09 | **2,35** | 7,099E-11 | **1,868** | 6,642E-09 |
| **TRIB2** | **ENSG00000071575** | **2,299** | 1,576E-07 | **2,204** | 1,651E-08 | **2,62** | 1,229E-09 |
| **UBASH3B** | **ENSG00000154127** | **2,204** | 1,078E-09 | **0,644** | 0,0017069 | **1,332** | 2,435E-07 |
| **LAMP3** | **ENSG00000078081** | **2,193** | 1,499E-05 | **2,505** | 1,69E-07 | **3,713** | 2,672E-10 |
| **AMIGO2** | **ENSG00000139211** | **2,175** | 1,036E-10 | **2,289** | 8,26E-13 | **2,149** | 4,013E-12 |
| **VEGFC** | **ENSG00000150630** | **2,144** | 4,369E-08 | **1,532** | 5,447E-07 | **2,119** | 3,701E-09 |
| **FOSL1** | **ENSG00000175592** | **2,109** | 3,79E-08 | **3,995** | 3,393E-14 | **2,456** | 1,87E-10 |
| **RAPGEF3** | **ENSG00000079337** | **1,998** | 6,57E-07 | **2,877** | 7,09E-11 | **0,986** | 0,0009229 |
| **DACT1** | **ENSG00000165617** | **1,985** | 2,907E-07 | **2,181** | 3,04E-09 | **3,068** | 1,34E-11 |
| **TGFA** | **ENSG00000163235** | **1,972** | 1,436E-06 | **1,769** | 5,589E-07 | **2,277** | 1,24E-08 |
| **IER3** | **ENSG00000137331** | **1,929** | 4,906E-12 | **1,345** | 2,772E-11 | **1,071** | 1,932E-09 |
| **HAS2** | **ENSG00000170961** | **1,924** | 1,039E-09 | **1,672** | 3,212E-10 | **1,695** | 4,424E-10 |
| **HMGA2** | **ENSG00000149948** | **1,916** | 8,512E-05 | **2,398** | 3,32E-07 | **3,124** | 5,38E-09 |
| **NT5E** | **ENSG00000135318** | **1,904** | 7,483E-08 | **1,333** | 9,657E-07 | **1,476** | 2,802E-07 |
| **SPRED2** | **ENSG00000198369** | **1,829** | 3,973E-13 | **1,099** | 2,091E-11 | **1,489** | 2,852E-13 |
| **ANTXR2** | **ENSG00000163297** | **1,807** | 6,697E-07 | **1,115** | 5,355E-05 | **1,547** | 7,47E-07 |
| **MICAL2** | **ENSG00000133816** | **1,784** | 1,751E-10 | **0,671** | 1,963E-05 | **0,625** | 7,131E-05 |
| **TLR4** | **ENSG00000136869** | **1,778** | 2,977E-10 | **3,295** | 2,85E-16 | **3,847** | 1,586E-16 |
| **KCNK2** | **ENSG00000082482** | **1,74** | 3,734E-07 | **0,788** | 0,0010368 | **1,939** | 4,977E-09 |
| **CLDN1** | **ENSG00000163347** | **1,68** | 1,434E-09 | **2,728** | 1,241E-14 | **3,257** | 2,526E-15 |
| **ETV1** | **ENSG00000006468** | **1,678** | 1,751E-10 | **0,922** | 8,327E-08 | **1,216** | 1,476E-09 |
| **SMOX** | **ENSG00000088826** | **1,657** | 1,208E-06 | **2,034** | 2,356E-09 | **1,707** | 7,137E-08 |
| **PTX3** | **ENSG00000163661** | **1,618** | 0,0087534 | **4,14** | 5,838E-09 | **4,527** | 1,688E-09 |
| **CXCL2** | **ENSG00000081041** | **1,612** | 2,293E-06 | **0,911** | 0,0003938 | **1,597** | 2,835E-07 |
| **CCND1** | **ENSG00000110092** | **1,608** | 1,971E-10 | **0,676** | 2,271E-06 | **1,065** | 3,669E-09 |
| **MRAP2** | **ENSG00000135324** | **1,592** | 1,192E-07 | **1,135** | 1,318E-06 | **2,168** | 4,166E-11 |
| **UNC13A** | **ENSG00000130477** | **1,587** | 7,957E-06 | **1,454** | 2,536E-06 | **0,618** | 0,0211111 |
| **KIAA0040** | **ENSG00000235750** | **1,587** | 3,973E-13 | **0,849** | 7,551E-11 | **1,041** | 5,33E-12 |
| **FZD8** | **ENSG00000177283** | **1,579** | 3,335E-07 | **0,92** | 5,633E-05 | **2,169** | 1,2E-10 |
| **LETM2** | **ENSG00000165046** | **1,575** | 1,973E-07 | **2,692** | 8,129E-13 | **1,921** | 5,271E-10 |
| **SERTAD4** | **ENSG00000082497** | **1,57** | 0,0238585 | **2,055** | 0,0008682 | **5,08** | 1,393E-09 |
| **IL4R** | **ENSG00000077238** | **1,547** | 3,238E-08 | **1,423** | 5,432E-09 | **1,133** | 3,432E-07 |
| **ENC1** | **ENSG00000171617** | **1,527** | 2,745E-08 | **2,369** | 6,652E-13 | **3,061** | 2,376E-14 |
| **ABLIM3** | **ENSG00000173210** | **1,511** | 1,216E-09 | **0,936** | 9,948E-08 | **1,976** | 7,596E-13 |
| **DUSP5** | **ENSG00000138166** | **1,496** | 6,186E-08 | **3,339** | 2,489E-15 | **3,96** | 6,124E-16 |
| **THBS1** | **ENSG00000137801** | **1,493** | 6,178E-09 | **0,58** | 0,0001087 | **1,704** | 4,087E-11 |
| **EREG** | **ENSG00000124882** | **1,491** | 3,723E-05 | **2,63** | 3,031E-10 | **2,303** | 4,909E-09 |
| **LAMC2** | **ENSG00000058085** | **1,487** | 0,00228 | **4,082** | 9,979E-11 | **3,469** | 2,53E-09 |
| **FST** | **ENSG00000134363** | **1,47** | 1,585E-05 | **3,53** | 4,425E-13 | **4,83** | 6,733E-15 |
| **TNFRSF21** | **ENSG00000146072** | **1,447** | 1,756E-11 | **0,947** | 2,521E-10 | **1,335** | 1,813E-12 |
| **NEDD9** | **ENSG00000111859** | **1,443** | 1,891E-06 | **1,421** | 1,741E-07 | **1,939** | 1,642E-09 |
| **AFAP1L2** | **ENSG00000169129** | **1,414** | 0,0180195 | **4,318** | 1,875E-09 | **2,767** | 3,831E-06 |
| **SLC16A6** | **ENSG00000108932** | **1,408** | 6,74E-05 | **1,608** | 1,08E-06 | **2,757** | 1,812E-10 |
| **ICAM1** | **ENSG00000090339** | **1,406** | 3,283E-09 | **2,282** | 2,681E-14 | **2,279** | 6,331E-14 |
| **ANKRD1** | **ENSG00000148677** | **1,401** | 0,0053562 | **2,084** | 1,288E-05 | **3,18** | 2,131E-08 |
| **TMEM52B** | **ENSG00000165685** | **1,385** | 0,0043481 | **4,343** | 3,541E-11 | **5,368** | 1,584E-12 |
| **IRAK2** | **ENSG00000134070** | **1,376** | 5,653E-08 | **2,015** | 3,352E-12 | **1,077** | 2,204E-07 |
| **FOSB** | **ENSG00000125740** | **1,342** | 0,0019413 | **3,644** | 8,829E-11 | **3,976** | 3,059E-11 |
| **ITGA4** | **ENSG00000115232** | **1,31** | 7,004E-07 | **0,715** | 0,0002043 | **1,452** | 1,163E-08 |
| **CCN2** | **ENSG00000118523** | **1,306** | 1,348E-05 | **1,26** | 2,25E-06 | **1,759** | 2,136E-08 |
| **F3** | **ENSG00000117525** | **1,287** | 1,513E-05 | **1,288** | 1,397E-06 | **2,4** | 9,908E-11 |
| **IL17RD** | **ENSG00000144730** | **1,274** | 8,486E-05 | **1,481** | 1,092E-06 | **2,289** | 1,175E-09 |
| **PADI1** | **ENSG00000142623** | **1,27** | 0,0008633 | **4,218** | 6,193E-13 | **3,706** | 1,017E-11 |
| **RGS2** | **ENSG00000116741** | **1,265** | 1,52E-10 | **1,1** | 2,529E-11 | **1,016** | 1,646E-10 |
| **PTGS2** | **ENSG00000073756** | **1,262** | 1,578E-06 | **1,599** | 2,022E-09 | **1,435** | 2,005E-08 |
| **JUN** | **ENSG00000177606** | **1,256** | 1,287E-05 | **1,347** | 4,199E-07 | **1,68** | 1,823E-08 |
| **EPHA2** | **ENSG00000142627** | **1,249** | 2,664E-10 | **2,498** | 1,704E-16 | **2,283** | 1,866E-15 |
| **RAB3B** | **ENSG00000169213** | **1,244** | 3,735E-07 | **1,745** | 5,775E-11 | **2,142** | 3,327E-12 |
| **SLC1A3** | **ENSG00000079215** | **1,22** | 0,0006305 | **2,095** | 3,684E-08 | **2,312** | 9,96E-09 |
| **EPAS1** | **ENSG00000116016** | **1,214** | 3,103E-08 | **0,533** | 0,0001518 | **1,755** | 5,505E-12 |
| **ANGPTL4** | **ENSG00000167772** | **1,21** | 0,0005691 | **2,375** | 3,173E-09 | **1,753** | 7,686E-07 |
| **STEAP1** | **ENSG00000164647** | **1,209** | 1,195E-05 | **1,478** | 4,189E-08 | **1,915** | 8,185E-10 |
| **DCLK1** | **ENSG00000133083** | **1,201** | 9,116E-06 | **0,639** | 0,0017423 | **0,533** | 0,0094727 |
| **FCMR** | **ENSG00000162894** | **1,176** | 2,277E-05 | **2,067** | 1,837E-10 | **2,547** | 9,002E-12 |
| **FOXL1** | **ENSG00000176678** | **1,175** | 3,264E-10 | **1,076** | 3,474E-11 | **0,927** | 7,836E-10 |
| **JAG1** | **ENSG00000101384** | **1,167** | 2,34E-08 | **1,605** | 4,447E-12 | **2,227** | 4,444E-14 |
| **LANCL3** | **ENSG00000147036** | **1,158** | 2,461E-05 | **0,744** | 0,0006046 | **1,487** | 7,253E-08 |
| **CITED4** | **ENSG00000179862** | **1,147** | 0,0002235 | **1,541** | 4,298E-07 | **1,684** | 1,55E-07 |
| **COX6B2** | **ENSG00000160471** | **1,141** | 6,256E-05 | **0,689** | 0,0021679 | **1,49** | 1,674E-07 |
| **TNFAIP6** | **ENSG00000123610** | **1,125** | 0,0001305 | **1,964** | 2,404E-09 | **2,195** | 5,591E-10 |
| **RHEBL1** | **ENSG00000167550** | **1,121** | 0,0001197 | **3,067** | 7,594E-13 | **3,702** | 6,244E-14 |
| **BMP4** | **ENSG00000125378** | **1,09** | 0,0131996 | **2,162** | 1,188E-06 | **2,387** | 3,373E-07 |
| **DKK1** | **ENSG00000107984** | **1,079** | 0,01511 | **4,813** | 2,457E-12 | **4,864** | 3,379E-12 |
| **STAMBPL1** | **ENSG00000138134** | **1,059** | 8,516E-07 | **1,04** | 6,862E-08 | **1,092** | 4,862E-08 |
| **HBEGF** | **ENSG00000113070** | **1,046** | 1,545E-06 | **2,277** | 1,621E-13 | **2,176** | 7,669E-13 |
| **TPBG** | **ENSG00000146242** | **1,03** | 5,441E-08 | **1,639** | 1,198E-12 | **1,866** | 2,836E-13 |
| **TMEM156** | **ENSG00000121895** | **1,025** | 0,00243 | **2,56** | 6,886E-10 | **2,524** | 1,399E-09 |
| **TMEM154** | **ENSG00000170006** | **1,015** | 0,0005166 | **2,341** | 1,787E-10 | **2,167** | 1,161E-09 |
| **DDX58** | **ENSG00000107201** | **1,015** | 1,605E-05 | **2,632** | 1,966E-13 | **4,135** | 5,659E-16 |
| **PTGER4** | **ENSG00000171522** | **1,014** | 3,796E-05 | **3,009** | 4,395E-14 | **2,948** | 1,336E-13 |
| **EDNRA** | **ENSG00000151617** | **1,012** | 1,973E-07 | **0,894** | 7,068E-08 | **1,143** | 2,149E-09 |
| **ERRFI1** | **ENSG00000116285** | **1,012** | 2,374E-08 | **1,966** | 1,31E-14 | **2,858** | 2,047E-16 |
| **AREG** | **ENSG00000109321** | **1,002** | 0,0064749 | **4,427** | 2,506E-13 | **3,973** | 3,009E-12 |
| **DRAM1** | **ENSG00000136048** | **1,001** | 4,237E-09 | **0,826** | 3,086E-09 | **0,608** | 4,262E-07 |
| **IRS1** | **ENSG00000169047** | **1** | 2,251E-09 | **0,899** | 3,684E-10 | **0,911** | 4,841E-10 |
| **MAFF** | **ENSG00000185022** | **0,998** | 0,0036363 | **3,325** | 1,16E-11 | **3,201** | 3,536E-11 |
| **TFPI2** | **ENSG00000105825** | **0,992** | 3,186E-09 | **1,801** | 5,408E-15 | **4,154** | 7,552E-20 |
| **SH3TC2** | **ENSG00000169247** | **0,99** | 0,0005627 | **2,558** | 2,815E-11 | **1,582** | 1,704E-07 |
| **RASGRP1** | **ENSG00000172575** | **0,988** | 0,0189583 | **0,941** | 0,0098333 | **4,044** | 2,327E-11 |
| **PDP1** | **ENSG00000164951** | **0,982** | 3,855E-08 | **1,592** | 5,253E-13 | **1,779** | 1,726E-13 |
| **CHAC1** | **ENSG00000128965** | **0,973** | 0,002171 | **2,279** | 1,844E-09 | **2,928** | 3,617E-11 |
| **IFIT2** | **ENSG00000119922** | **0,972** | 0,0058829 | **3,143** | 7,404E-11 | **4,742** | 1,661E-13 |
| **PLK3** | **ENSG00000173846** | **0,963** | 1,145E-05 | **3,058** | 3,601E-15 | **2,361** | 5,521E-13 |
| **PRSS23** | **ENSG00000150687** | **0,959** | 1,138E-06 | **1,523** | 6,167E-11 | **1,968** | 1,908E-12 |
| **ADM2** | **ENSG00000128165** | **0,954** | 0,0006544 | **0,623** | 0,0067198 | **2,125** | 7,96E-10 |
| **TINAGL1** | **ENSG00000142910** | **0,949** | 8,31E-05 | **1,884** | 2,416E-10 | **2,619** | 1,898E-12 |
| **ITGA2** | **ENSG00000164171** | **0,948** | 2,101E-06 | **0,674** | 1,886E-05 | **1,12** | 1,607E-08 |
| **FOXO1** | **ENSG00000150907** | **0,937** | 0,0005186 | **0,769** | 0,0007526 | **2,193** | 2,325E-10 |
| **CORO1A** | **ENSG00000102879** | **0,933** | 0,0180769 | **3,324** | 1,545E-10 | **2,045** | 9,092E-07 |
| **PRAG1** | **ENSG00000275342** | **0,929** | 8,555E-07 | **1,626** | 6,07E-12 | **2,129** | 1,462E-13 |
| **TNFRSF1B** | **ENSG00000028137** | **0,915** | 0,0076806 | **1,493** | 7,485E-06 | **2,113** | 4,635E-08 |
| **GBX2** | **ENSG00000168505** | **0,913** | 0,0255982 | **2,636** | 1,67E-08 | **2,443** | 8,474E-08 |
| **RELB** | **ENSG00000104856** | **0,904** | 0,0137498 | **4,246** | 8,915E-13 | **2,796** | 1,393E-09 |
| **CREM** | **ENSG00000095794** | **0,883** | 1,535E-06 | **1,175** | 9,21E-10 | **1,499** | 2,727E-11 |
| **RAET1E** | **ENSG00000164520** | **0,882** | 1,326E-07 | **0,671** | 4,33E-07 | **1,026** | 8,809E-10 |
| **ARHGDIB** | **ENSG00000111348** | **0,877** | 0,000393 | **2,039** | 1,006E-10 | **1,402** | 1,046E-07 |
| **SMAGP** | **ENSG00000170545** | **0,874** | 0,0006269 | **1,517** | 3,558E-08 | **0,738** | 0,0009248 |
| **NFE2L3** | **ENSG00000050344** | **0,864** | 0,0002596 | **1,208** | 3,015E-07 | **1,456** | 2,26E-08 |
| **PLK2** | **ENSG00000145632** | **0,863** | 3,335E-06 | **2,124** | 2,304E-13 | **1,991** | 1,087E-12 |
| **PLAU** | **ENSG00000122861** | **0,862** | 0,0031709 | **2,598** | 5,091E-11 | **2,239** | 1,056E-09 |
| **TMEM37** | **ENSG00000171227** | **0,861** | 0,0001522 | **1,391** | 1,293E-08 | **2,178** | 9,82E-12 |
| **STING1** | **ENSG00000184584** | **0,844** | 3,103E-08 | **2,585** | 2,071E-17 | **2,022** | 2,773E-15 |
| **IL7R** | **ENSG00000168685** | **0,839** | 0,0002147 | **2,112** | 4,044E-11 | **2,882** | 5,417E-13 |
| **OASL** | **ENSG00000135114** | **0,808** | 0,0101706 | **2,167** | 5,652E-09 | **5,193** | 7,188E-15 |
| **PLAUR** | **ENSG00000011422** | **0,805** | 2,34E-08 | **0,639** | 3,431E-08 | **0,719** | 8,214E-09 |
| **PLEK2** | **ENSG00000100558** | **0,802** | 3,901E-07 | **1,323** | 4,71E-12 | **1,006** | 8,549E-10 |
| **PTPRR** | **ENSG00000153233** | **0,8** | 0,0476753 | **2,876** | 3,301E-09 | **2,919** | 3,785E-09 |
| **SLFN5** | **ENSG00000166750** | **0,797** | 1,048E-06 | **2,07** | 1,258E-14 | **3,511** | 2,423E-17 |
| **CASP4** | **ENSG00000196954** | **0,796** | 1,594E-05 | **2,201** | 6,127E-14 | **1,428** | 1,826E-10 |
| **ZSWIM4** | **ENSG00000132003** | **0,791** | 5,26E-05 | **1,648** | 5,09E-11 | **1,986** | 4,179E-12 |
| **TNFRSF12A** | **ENSG00000006327** | **0,789** | 3,02E-07 | **1,139** | 5,311E-11 | **0,514** | 9,539E-06 |
| **TGFBR2** | **ENSG00000163513** | **0,773** | 1,19E-08 | **0,59** | 3,071E-08 | **0,671** | 5,91E-09 |
| **TOR4A** | **ENSG00000198113** | **0,756** | 9,443E-07 | **1,283** | 1,352E-11 | **0,523** | 1,468E-05 |
| **CMTM3** | **ENSG00000140931** | **0,737** | 0,0001064 | **1,809** | 7,557E-12 | **0,89** | 1,365E-06 |
| **SYNE3** | **ENSG00000176438** | **0,73** | 0,0015629 | **0,993** | 7,004E-06 | **1,922** | 2,545E-10 |
| **PI3** | **ENSG00000124102** | **0,728** | 0,03203 | **3,715** | 1,853E-12 | **3,298** | 2,391E-11 |
| **SPATA2L** | **ENSG00000158792** | **0,724** | 0,0005627 | **1,191** | 7,928E-08 | **1,207** | 1,004E-07 |
| **FGFR1** | **ENSG00000077782** | **0,713** | 1,11E-06 | **1,122** | 4,585E-11 | **2,183** | 3,081E-15 |
| **PTGES** | **ENSG00000148344** | **0,71** | 0,0034042 | **2,487** | 1,999E-11 | **1,607** | 2,911E-08 |
| **OAS1** | **ENSG00000089127** | **0,708** | 0,0006165 | **1,474** | 2,333E-09 | **2,424** | 1,069E-12 |
| **SH2B3** | **ENSG00000111252** | **0,704** | 0,0001088 | **1,309** | 8,771E-10 | **0,605** | 0,0001259 |
| **HES1** | **ENSG00000114315** | **0,697** | 0,0001463 | **1,461** | 1,551E-10 | **1,408** | 5,262E-10 |
| **C3** | **ENSG00000125730** | **0,697** | 0,0001574 | **0,502** | 0,00079 | **2,164** | 2,169E-12 |
| **TRANK1** | **ENSG00000168016** | **0,696** | 3,723E-05 | **2,548** | 2,509E-15 | **2,869** | 1,487E-15 |
| **RGS16** | **ENSG00000143333** | **0,695** | 0,0044755 | **1,257** | 6,761E-07 | **2,325** | 3,267E-11 |
| **STX11** | **ENSG00000135604** | **0,689** | 0,0158004 | **3,175** | 1,463E-12 | **2,703** | 4,341E-11 |
| **SLC26A2** | **ENSG00000155850** | **0,689** | 1,779E-06 | **0,849** | 5,556E-09 | **0,574** | 2,226E-06 |
| **SHROOM3** | **ENSG00000138771** | **0,682** | 2,597E-08 | **0,78** | 1,163E-10 | **1,052** | 1,827E-12 |
| **KLRC3** | **ENSG00000205810** | **0,679** | 0,0111091 | **1,265** | 2,825E-06 | **2,764** | 9,014E-12 |
| **FRMD6** | **ENSG00000139926** | **0,651** | 1,507E-05 | **0,855** | 2,313E-08 | **0,622** | 3,671E-06 |
| **GLRX** | **ENSG00000173221** | **0,648** | 1,193E-05 | **1,633** | 3,154E-13 | **2,151** | 9,641E-15 |
| **CD3EAP** | **ENSG00000117877** | **0,645** | 7,885E-06 | **0,719** | 1,461E-07 | **0,614** | 1,829E-06 |
| **PPP1R3B** | **ENSG00000173281** | **0,644** | 0,000148 | **1,502** | 2,518E-11 | **1,17** | 3,388E-09 |
| **AHR** | **ENSG00000106546** | **0,64** | 7,661E-07 | **0,861** | 4,788E-10 | **1,295** | 1,31E-12 |
| **MEAK7** | **ENSG00000140950** | **0,628** | 1,61E-06 | **0,984** | 7,468E-11 | **0,688** | 4,065E-08 |
| **GNG2** | **ENSG00000186469** | **0,625** | 0,004027 | **1,495** | 5,858E-09 | **0,747** | 0,0002214 |
| **SDC4** | **ENSG00000124145** | **0,625** | 2,798E-06 | **2,134** | 9,655E-16 | **1,804** | 2,886E-14 |
| **PKP2** | **ENSG00000057294** | **0,624** | 0,0014235 | **2,021** | 4,481E-12 | **1,927** | 1,861E-11 |
| **DUSP23** | **ENSG00000158716** | **0,622** | 9,106E-05 | **1,154** | 6,735E-10 | **1,132** | 1,7E-09 |
| **AFAP1L1** | **ENSG00000157510** | **0,616** | 0,0003088 | **1,291** | 5,884E-10 | **1,117** | 1,121E-08 |
| **CAPRIN2** | **ENSG00000110888** | **0,615** | 8,138E-07 | **0,887** | 1,197E-10 | **1,017** | 2,397E-11 |
| **PLAT** | **ENSG00000104368** | **0,609** | 0,0023476 | **1,722** | 1,088E-10 | **0,892** | 7,381E-06 |
| **SAT1** | **ENSG00000130066** | **0,607** | 0,0002878 | **2,217** | 4,846E-14 | **2,968** | 1,66E-15 |
| **LGR6** | **ENSG00000133067** | **0,602** | 0,0230044 | **1,65** | 3,826E-08 | **2,805** | 7,359E-12 |
| **FOS** | **ENSG00000170345** | **0,597** | 0,0017317 | **1,512** | 4,2E-10 | **2,338** | 4,457E-13 |
| **EMILIN2** | **ENSG00000132205** | **0,596** | 0,0134408 | **1,45** | 6,85E-08 | **1,883** | 1,275E-09 |
| **PRDM1** | **ENSG00000057657** | **0,59** | 0,0162797 | **2,181** | 8,983E-11 | **1,434** | 1,728E-07 |
| **PHLDA2** | **ENSG00000181649** | **0,585** | 0,0023032 | **1,807** | 4,09E-11 | **1,551** | 8,276E-10 |
| **XK** | **ENSG00000047597** | **0,582** | 0,0019508 | **0,778** | 1,319E-05 | **0,923** | 1,835E-06 |
| **NKX3-1** | **ENSG00000167034** | **0,581** | 0,0027151 | **2,218** | 8,316E-13 | **2,136** | 3,168E-12 |
| **C6orf141** | **ENSG00000197261** | **0,576** | 0,001725 | **0,772** | 1,048E-05 | **1,492** | 5,168E-10 |
| **PTGS1** | **ENSG00000095303** | **0,571** | 0,0116758 | **1,389** | 5,26E-08 | **0,941** | 2,577E-05 |
| **ARL4C** | **ENSG00000188042** | **0,569** | 0,0168305 | **1,615** | 9,705E-09 | **1,753** | 3,802E-09 |
| **ZNF774** | **ENSG00000196391** | **0,568** | 0,0021884 | **1,633** | 7,144E-11 | **1,448** | 1,064E-09 |
| **CCN3** | **ENSG00000136999** | **0,568** | 0,0030336 | **1,478** | 1,665E-09 | **2,273** | 2,514E-12 |
| **TNFRSF10A** | **ENSG00000104689** | **0,562** | 5,328E-05 | **1,241** | 2,655E-11 | **1,096** | 3,346E-10 |
| **MMD** | **ENSG00000108960** | **0,559** | 0,0002666 | **1,276** | 1,031E-10 | **0,793** | 4,587E-07 |
| **SNAPC1** | **ENSG00000023608** | **0,559** | 0,003096 | **0,969** | 7,861E-07 | **1,88** | 2,731E-11 |
| **RGL2** | **ENSG00000237441** | **0,558** | 2,547E-05 | **0,667** | 1,856E-07 | **1,381** | 2,866E-12 |
| **GATA6** | **ENSG00000141448** | **0,556** | 0,0001471 | **0,726** | 4,472E-07 | **1,015** | 3,676E-09 |
| **HYI** | **ENSG00000178922** | **0,553** | 2,511E-05 | **0,77** | 1,697E-08 | **0,714** | 8,429E-08 |
| **STK17A** | **ENSG00000164543** | **0,553** | 0,0008673 | **0,966** | 1,023E-07 | **1,225** | 3,438E-09 |
| **NFATC1** | **ENSG00000131196** | **0,545** | 0,0052322 | **0,535** | 0,0016033 | **0,762** | 5,138E-05 |
| **PLPP3** | **ENSG00000162407** | **0,542** | 0,0002538 | **0,868** | 4,11E-08 | **1,504** | 8,151E-12 |
| **NOCT** | **ENSG00000151014** | **0,539** | 0,0012564 | **1,348** | 3,576E-10 | **1,993** | 9,397E-13 |
| **ULBP3** | **ENSG00000131019** | **0,539** | 0,0035405 | **1,646** | 7,784E-11 | **0,74** | 3,491E-05 |
| **ZNF41** | **ENSG00000147124** | **0,537** | 1,54E-06 | **0,561** | 5,287E-08 | **0,798** | 3,181E-10 |
| **SLC37A3** | **ENSG00000157800** | **0,533** | 1,559E-05 | **1,136** | 7,781E-12 | **0,812** | 3,43E-09 |
| **BHLHE41** | **ENSG00000123095** | **0,528** | 0,0443032 | **1,698** | 2,877E-08 | **3,939** | 2,588E-14 |
| **LONRF3** | **ENSG00000175556** | **0,523** | 1,351E-05 | **0,582** | 2,375E-07 | **0,779** | 4,102E-09 |
| **ARID5A** | **ENSG00000196843** | **0,521** | 0,0030381 | **1,235** | 4,217E-09 | **1,401** | 8,64E-10 |
| **GNG11** | **ENSG00000127920** | **0,519** | 1,085E-07 | **0,888** | 1,304E-12 | **1,35** | 5,701E-15 |
| **ARHGEF5** | **ENSG00000050327** | **0,518** | 0,0041846 | **0,958** | 4,568E-07 | **0,621** | 0,0002267 |
| **TCIM** | **ENSG00000176907** | **0,517** | 0,002951 | **0,888** | 1,315E-06 | **1,385** | 2,197E-09 |
| **HTR1D** | **ENSG00000179546** | **0,516** | 0,0216213 | **0,8** | 0,0001463 | **1,63** | 7,75E-09 |
| **DDIAS** | **ENSG00000165490** | **0,515** | 5,389E-06 | **1,145** | 1,449E-12 | **1,269** | 5,417E-13 |
| **POPDC3** | **ENSG00000132429** | **0,512** | 0,009784 | **1,385** | 5,941E-09 | **0,808** | 3,383E-05 |
| **SERPINB8** | **ENSG00000166401** | **0,509** | 0,0005861 | **1,435** | 1,331E-11 | **1,348** | 7,148E-11 |
| **ZNF217** | **ENSG00000171940** | **0,509** | 3,801E-06 | **0,844** | 1,21E-10 | **1,071** | 4,704E-12 |
| **PKIA** | **ENSG00000171033** | **0,508** | 0,0171343 | **0,501** | 0,0066219 | **1,221** | 2,709E-07 |
| **CYSRT1** | **ENSG00000197191** | **0,504** | 0,0306707 | **2,485** | 4,432E-12 | **2,42** | 1,221E-11 |
| **CCNYL1** | **ENSG00000163249** | **0,503** | 1,899E-05 | **1,002** | 3,724E-11 | **1,121** | 1,109E-11 |
| **MCL1** | **ENSG00000143384** | **0,503** | 3,635E-09 | **0,767** | 1,889E-13 | **0,822** | 1,213E-13 |

**Table S2**.

List of genes listed in table S1 upregulated in small cells.

| Gene.name | Row.names | ratio Small/Big | FDR |
| --- | --- | --- | --- |
| LGR6 | ENSG00000133067 | 3,262756358 | 1,99223E-29 |
| PKP2 | ENSG00000057294 | 1,956376143 | 1,77426E-14 |
| KLRC3 | ENSG00000205810 | 1,858171662 | 0,001700306 |
| TNFRSF1B | ENSG00000028137 | 1,800508591 | 0,008155063 |
| KIAA0040 | ENSG00000235750 | 1,742336558 | 1,77664E-23 |
| RASGRP1 | ENSG00000172575 | 1,713797902 | 0,022009742 |
| UNC13A | ENSG00000130477 | 1,686885031 | 0,003256944 |
| RGS16 | ENSG00000143333 | 1,668132852 | 1,95835E-06 |
| GATA6 | ENSG00000141448 | 1,603586543 | 3,24696E-06 |
| FOXO1 | ENSG00000150907 | 1,596991433 | 0,000663545 |
| TOR4A | ENSG00000198113 | 1,586528482 | 1,34775E-07 |
| IL6 | ENSG00000136244 | 1,576825826 | 2,49034E-12 |
| DUSP4 | ENSG00000120875 | 1,5650394 | 4,07321E-15 |
| CXCL1 | ENSG00000163739 | 1,557967804 | 1,34969E-06 |
| NFATC1 | ENSG00000131196 | 1,551655624 | 4,00685E-05 |
| ANGPTL4 | ENSG00000167772 | 1,5375302 | 0,005742422 |
| RELB | ENSG00000104856 | 1,511293276 | 0,001090686 |
| TNFSF15 | ENSG00000181634 | 1,461296383 | 0,020848507 |
| OAS1 | ENSG00000089127 | 1,439470079 | 2,29959E-05 |
| FZD8 | ENSG00000177283 | 1,413876636 | 0,000425811 |
| CXCL8 | ENSG00000169429 | 1,408556685 | 1,83334E-07 |
| PHLDA2 | ENSG00000181649 | 1,383762162 | 0,025921981 |
| DUSP23 | ENSG00000158716 | 1,375591224 | 0,00138809 |
| ICAM1 | ENSG00000090339 | 1,372786131 | 0,00022572 |
| STX11 | ENSG00000135604 | 1,358803609 | 0,011618697 |
| SHROOM3 | ENSG00000138771 | 1,335481411 | 1,1047E-05 |
| PLAU | ENSG00000122861 | 1,327970117 | 0,03463661 |
| FOSB | ENSG00000125740 | 1,31353545 | 4,79733E-06 |
| FOXL1 | ENSG00000176678 | 1,307994291 | 0,000259727 |
| NKX3-1 | ENSG00000167034 | 1,297237939 | 0,004824138 |
| CXCL2 | ENSG00000081041 | 1,289978458 | 0,000183427 |
| XK | ENSG00000047597 | 1,282982283 | 0,014527829 |
| SMOX | ENSG00000088826 | 1,281381747 | 0,047193001 |
| IL4R | ENSG00000077238 | 1,275718256 | 0,008193769 |
| CD3EAP | ENSG00000117877 | 1,273461886 | 0,000106572 |
| JUN | ENSG00000177606 | 1,230391924 | 0,000787966 |
| IRAK2 | ENSG00000134070 | 1,227564669 | 0,006415166 |
| NT5E | ENSG00000135318 | 1,221850797 | 0,011170514 |
| LONRF3 | ENSG00000175556 | 1,220475282 | 0,013971681 |
| PRAG1 | ENSG00000275342 | 1,21610736 | 0,014809852 |
| IRS1 | ENSG00000169047 | 1,202671356 | 0,023223375 |
| ENC1 | ENSG00000171617 | 1,199259496 | 0,01402304 |
| JAG1 | ENSG00000101384 | 1,197858272 | 0,002770563 |
| SAT1 | ENSG00000130066 | 1,182262647 | 0,014284228 |
| IER3 | ENSG00000137331 | 1,170411242 | 0,022790622 |
| FOS | ENSG00000170345 | 1,166117283 | 0,022260792 |

**Table S3**.

List of genes listed in table S1 downregulated in small cells.

| Gene.name | Row.names | **ratio Small/Big** | FDR |
| --- | --- | --- | --- |
| ANKRD1 | ENSG00000148677 | **0,34258976** | 1,8602E-23 |
| SLC16A6 | ENSG00000108932 | **0,49208769** | 8,9629E-15 |
| NGF | ENSG00000134259 | **0,5181317** | 0,00057277 |
| POPDC3 | ENSG00000132429 | **0,52178412** | 8,196E-06 |
| ITGB3 | ENSG00000259207 | **0,52326105** | 3,7834E-06 |
| STEAP1 | ENSG00000164647 | **0,54925844** | 0,0001026 |
| FST | ENSG00000134363 | **0,56887095** | 5,0506E-11 |
| EREG | ENSG00000124882 | **0,5830599** | 1,0015E-15 |
| PPP1R3B | ENSG00000173281 | **0,59796553** | 2,4996E-11 |
| MICAL2 | ENSG00000133816 | **0,60258937** | 4,7744E-11 |
| ABLIM3 | ENSG00000173210 | **0,61637525** | 1,6851E-07 |
| AMIGO2 | ENSG00000139211 | **0,65946717** | 5,5239E-12 |
| ANTXR2 | ENSG00000163297 | **0,70044795** | 0,03060774 |
| SLC1A3 | ENSG00000079215 | **0,71089643** | 0,01098708 |
| PADI1 | ENSG00000142623 | **0,71632814** | 0,02812852 |
| SH3TC2 | ENSG00000169247 | **0,71828216** | 0,02222198 |
| ITGA4 | ENSG00000115232 | **0,71909755** | 0,00103833 |
| PRDM1 | ENSG00000057657 | **0,7318984** | 0,01323835 |
| STK17A | ENSG00000164543 | **0,73502172** | 1,1347E-06 |
| CMTM3 | ENSG00000140931 | **0,73516996** | 0,00694145 |
| PTPRR | ENSG00000153233 | **0,73634449** | 0,03280837 |
| C6orf141 | ENSG00000197261 | **0,74730583** | 0,00172687 |
| F3 | ENSG00000117525 | **0,75616217** | 1,6212E-06 |
| SH2B3 | ENSG00000111252 | **0,77451825** | 0,03060511 |
| HTR1D | ENSG00000179546 | **0,79285243** | 0,02172951 |
| ERRFI1 | ENSG00000116285 | **0,82224819** | 0,00214275 |
| FRMD6 | ENSG00000139926 | **0,82509974** | 0,00516706 |
| CASP4 | ENSG00000196954 | **0,82675415** | 0,03060511 |
| DDIAS | ENSG00000165490 | **0,83610323** | 0,03075383 |
| NEDD9 | ENSG00000111859 | **0,84426521** | 0,02948666 |
| RGS2 | ENSG00000116741 | **0,84671907** | 0,04319971 |

**References:**

1. GE, N. *et al.* Excessive Cell Growth Causes Cytoplasm Dilution And Contributes to Senescence. *Cell* **176**, 1083-1097.e18 (2019).

2. FJ, I. & V, B. Wide confocal cytometry: a new approach to study proteomic and structural changes in the cell nucleus during the cell cycle. *Histochem. Cell Biol.* **129**, 45–53 (2008).

3. Dobin, A. *et al.* STAR: Ultrafast universal RNA-seq aligner. *Bioinformatics* **29**, 15–21 (2013).

4. Kim, D., Paggi, J. M., Park, C., Bennett, C. & Salzberg, S. L. Graph-based genome alignment and genotyping with HISAT2 and HISAT-genotype. *Nat. Biotechnol.* **37**, 907–915 (2019).

5. Anders, S., Pyl, P. T. & Huber, W. HTSeq--a Python framework to work with high-throughput sequencing data. *Bioinformatics* **31**, 166–169 (2015).

6. Robinson, M. D., McCarthy, D. J. & Smyth, G. K. edgeR: a Bioconductor package for differential expression analysis of digital gene expression data. *Bioinformatics* **26**, 139–140 (2010).

7. Ritchie, M. E. *et al.* limma powers differential expression analyses for RNA-sequencing and microarray studies. *Nucleic Acids Res.* **43**, e47 (2015).

8. Yu, G., Wang, L. G., Han, Y. & He, Q. Y. clusterProfiler: an R package for comparing biological themes among gene clusters. *OMICS* **16**, 284–287 (2012).

9. Demidenko, E. & Miller, T. W. Statistical determination of synergy based on Bliss definition of drugs independence. *PLoS One* **14**, (2019).

10. Krumschnabel, G. *et al.* Simultaneous high-resolution measurement of mitochondrial respiration and hydrogen peroxide production. *Methods Mol. Biol.* **1264**, 245–261 (2015).
